# Research Progress on the Positive and Negative Regulatory Effects of Rhein on the Kidney: A Review of Its Molecular Targets

**DOI:** 10.3390/molecules27196572

**Published:** 2022-10-04

**Authors:** Yanna Zhu, Shilei Yang, Linlin Lv, Xiaohan Zhai, Guoyu Wu, Xiaolin Qi, Deshi Dong, Xufeng Tao

**Affiliations:** Department of Pharmacy, First Affiliated Hospital of Dalian Medical University, Dalian 116041, China

**Keywords:** traditional Chinese medicine, rhein, molecular targets, kidney protection, nephrotoxicity

## Abstract

Currently, both acute kidney injury (AKI) and chronic kidney disease (CKD) are considered to be the leading public health problems with gradually increasing incidence rates around the world. Rhein is a monomeric component of anthraquinone isolated from rhubarb, a traditional Chinese medicine. It has anti-inflammation, anti-oxidation, anti-apoptosis, anti-bacterial and other pharmacological activities, as well as a renal protective effects. Rhein exerts its nephroprotective effects mainly through decreasing hypoglycemic and hypolipidemic, playing anti-inflammatory, antioxidant and anti-fibrotic effects and regulating drug-transporters. However, the latest studies show that rhein also has potential kidney toxicity in case of large dosages and long use times. The present review highlights rhein’s molecular targets and its different effects on the kidney based on the available literature and clarifies that rhein regulates the function of the kidney in a positive and negative way. It will be helpful to conduct further studies on how to make full use of rhein in the kidney and to avoid kidney damage so as to make it an effective kidney protection drug.

## 1. Introduction

The kidney is an important organ in our body, and its main functions are to maintain fluid balance, regulate blood pressure, erythropoiesis, bone mineral density and hormone balance and to filter and remove nitrogen-containing and other waste products [1]. Microanatomic differences in perfusion, blood flow, and oxygen consumption make the kidneys more easily exposed to drugs, xenobiotics, and chemicals, which makes them more likely to be toxic targets [2]. The Global Burden of Disease 2015 study also indicates that nearly 1.2 million deaths are caused by kidney failure, and the number increased by 32% from 2005 to 2015 [3]. The GlomMS-II and transnational databases indicate that about 8 to 18% or 22% of hospitalized patients suffer from acute kidney injury (AKI) [4]. There is a sudden decline in renal function and a lower glomerular filtration rate (GFR) with elevated serum creatinine and blood urea nitrogen in AKI patients [5]. Drug induced kidney damage is common, and it primarily focused on AKI. A prospective cohort study associated with AKI due to drug exposure showed nephrotoxicity rates were about 14 to 26% in adults and 16% in the pediatric population in hospital [6]. Without early detection or treatment, AKI will develop into chronic kidney disease (CKD). Around the world, between 8% and 16% of people suffer from CKD, but most patients and clinicians are unaware of its implications [7,8]. Risk factors for CKD include prior exposure to potential nephrotoxins, history of nephrolithiasis or recurrent urinary tract infections, comorbidities, a family history of kidney disease, and other known genetic risk factors such as sickle cell trait [9,10,11]. Moreover, both AKI and CKD are considered to be the leading public health problems with gradually increasing incidence rates around the world [12].

Clinically, a few drugs have renal protective effects. There are three drug classes that exert nephroprotective effects [13]: renin-angiotensin-system inhibitors (RASi), vasopressin-receptor antagonists (VRA) and vaptans [14], and sodium–glucose transporter 2 inhibitors (SGLT2i) [15]. By preventing maladaptive glomerular hyperfiltration caused by circulating immunological factors, RASi, SGLT2i, and VRA delay kidney disease progression [13]. However, they can also cause a variety of side effects, for example, the adverse events commonly observed with tolvaptan [14]. The risks and benefits of using the above drugs must be carefully weighed. In traditional medicines, plants have a variety of pharmacological activities with lower toxicity, such as anti-inflammatory, antioxidant, anti-apoptotic, anti-fibrosis, and anti-cancer effects [1]. With the continuous progress of research, traditional Chinese medicine (TCM) has also shown gratifying results in kidney protection. Nephroprotective properties of phytoconstituents such as flavonoids, terpenoids, steroids, and fatty acids have been extensively investigated. Based on their ability to target mechanisms by modulating oxidative, inflammatory, and apoptotic factors, they are commonly used to treat kidney-related disorders [16].

Rhein (molecular formula C_15_H_8_O_6_), a lipophilic anthraquinone, is the main component of *Senna alexandrina* Mill., *Rheum palmatum* L., *Aloe barbadensis* Miller, and *Polygonum multiflorum* Thunb [17]. It contains two hydroxyl groups and one carboxyl group and has strong polarity and electrochemical REDOX properties [18]. Rhein has a lot of pharmacological effects, such as anti-inflammation [19], anti-cancer [20], anti-fibrosis [21], antioxidation [22], hepatoprotective [23], nephroprotective [24], lipid-lowering [25], and antimicrobial activities [26]. In spite of this, its poor solubility and low bioavailability limit its clinical applications. The study of rhein and its derivatives has been enriched by advances in drug separation and synthesis. Diacerein is one of the most common and representative derivatives of rhein, and it is used for the treatment of arthritis owing to its ability in reducing osteoclast formation and inhibiting the synthesis of resorptive factors [27]. Additionally, nanodrug delivery systems have been designed to overcome the poor solubility of rhein [28]. The pharmacological effects of these compounds lay the groundwork for the treatment of liver disease, osteoarthritis, diabetes, atherosclerosis, and a variety of cancers [29,30,31,32,33]. However, it has recently been reported that rhein also causes hepatotoxicity and nephrotoxicity [22,34]. This review summarizes and discusses the available literature related to the positive and negative regulatory effects of rhein on the kidney and shines a light on the use of rhein in kidney protection and the avoidance of kidney damage.

## 2. Nephroprotective Effect

**Figure 1:**
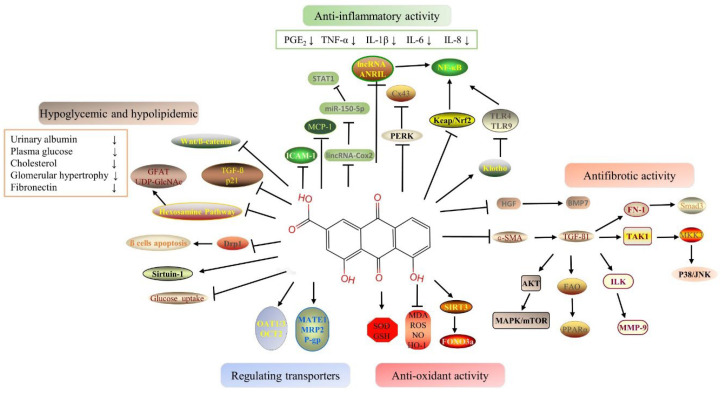
Signal pathway of the nephropropective effects of rhein.

The pharmacological benefits of rhein for human health are increasingly recognized. Rhein, and its derivatives, analogs, and compound preparations show the nephroprotective activities against various kidney disease, especially diabetic nephropathy (DN) and drug-induced AKI. One of the most common microvascular complications of diabetes is DN [35], which is a leading cause of CKD and end-stage renal disease (ESRD) worldwide [36]. The pathophysiology of DN is quite complex with two main initiating factors: abnormal metabolism and hemodynamic activity [37]. Pathogenesis of DN includes metabolic dysregulation, inflammation, oxidative stress, abnormal cytokines, etc., [38]. Considering multiple pathogenic mechanisms, we therefore need to develop drugs with multiple targets to treat DN effectively. The pathogenesis of AKI may be related to the specific injury of renal vessels, glomeruli, renal tubules, or interstitial compartments [2]. AKI can cause apoptosis, autophagy, and regulated and genetically controlled cell death as a result of cell damage [39]. Rhein has a variety of therapeutic targets as a TCM ingredient (or element), and its protective effect on the above targets has been gradually examined. Rhein is highly effective for the treatment of kidney-related diseases among natural components. The nephroprotective effects of rhein, rhein derivatives, and compound preparations are shown in Table 1, and the schematic mechanism of the signal pathway of the nephroprotective effects of rhein is shown in Figure 1.

### 2.1. Hypoglycemic and Hypolipidemic Proprieties of Rhein

Some studies have shown that the mitochondrial event chain caused by hyperglycemia may be one of the pathogeneses of diabetic nephropathy [78]. Rhein treatment showed significant improvements in glucose-dependent and independent insulin secretion in db/db mice by preserving β-cell mass and inhibiting apoptosis [45]. Another study [47] showed that rhein protects pancreatic cells from apoptosis by inhibiting the expression of dynamin-related protein 1 (Drp1). It may be possible to prevent or treat diabetes by rhein in the near future. Rhein has been shown to improve insulin resistance and renal injury in rats and db/db mice [79]. Furthermore, rhein and benazepril were evaluated individually and in combination in diabetic mice. Benazepril and rhein showed similar kidney protection [40]. In db/db mice, a combined application had a better kidney protection effect [40]. This reduced urinary albumin excretion, decreased plasma glucose, cholesterol, relieved glomerular hypertrophy, mesangial expansion and proliferation, and inhibited the expression of fibronectin (FN) and transforming growth factor- β1 (TGF-β1) [40]. In order to elucidate the therapeutic mechanism of rhein on DN, Zheng J et al. (2008) examined the effect of rhein on the hexosamine pathway [41]. Due to its role as a nutrient sensor, the hexosamine pathway has been associated with metabolic disorders and cellular hypertrophy when glucose levels are high [80]. The hexosamine pathway is one of the mechanisms responsible for renal damage in diabetes. To mimic mesangial cells under diabetic conditions, transgenic mesangial cell lines (MCGT1) overexpressing glucose transporter 1 (GLUT1) were used. GLUT1, an integral membrane protein, transports glucose into mesangial cells via glucose gradients [81]. In a medium containing 8 mM glucose, MCGT1 cells showed significant hypertrophy and synthesized much more FN. A 24 h treatment with rhein (25 μg/mL) significantly reduced MCGT1 hypertrophy and FN synthesis [81]. Firstly, rhein reduced glucose uptake by about 20%. Secondly, rhein markedly decreased glutamine, fructose 6-phosphate aminotransferase (GFAT) activity, the first and rate-limiting enzyme of the hexosamine pathway, and UDP-GlcNAc levels. Finally, rhein decreased TGF-β1 and p21 expression, which contributed to the decreased cellular hypertrophy and ECM synthesis in MCGT1 cells [81]. Decreasing lipidemia plays an important role in DN treatment [82]. Statins can reduce glomerular hypertension and directly protect renal inherent cells [83]. Using simvastatin as the control, Gao Q et al. (2010) investigated the effects of rhein on kidney damage and dyslipidemia in db/db mice with DN [42]. Rhein (150 mg/kg/d) is more effective than simvastatin in the reduction in albuminuria, TGF-β, and FN expression, and the ameliorations of obesity, plasma glucose, glomerular hypertrophy, and ECM expansion [42]. Meanwhile, rhein has a similar effect as simvastatin on dyslipidemia. Silent information regulator 2 homolog 1 (sirtuin-1) protects the kidney by modulating metabolic homeostasis, autophagy, oxidative stress, and inflammation [84]. In type 2 diabetic rats, rhein ameliorates renal injury by improving insulin resistance and dyslipidemia, as well as increasing SIRT1 (Sirtuin 1) expression [57]. Studies have shown that rhein has a nephroprotective effect on DN, which is also related to the Wnt/β-catenin signaling pathway. Activation of the Wnt/β-catenin signaling pathway may lead to proteinuria and kidney injury [59]. An immunofluorescence assay showed that the protein levels of nephrin, a podocyte specific marker, were significantly decreased in DN mice, while the protein levels of wnt1 and p-β-catenin were increased [59]. Rhein can reduce the thickness of the glomerular base and improve the histopathological changes in the kidney by regulating the Wnt/β-catenin signaling pathway [59].

### 2.2. Anti-Inflammatory Proprieties of Rhein

As a result of hyperglycemia, advanced glycation end products (AGEs) damage the vascular endothelium, glomeruli, and tubules, which facilitates the emergence of DN eventually [85]. Diabetes is an entity of inflammation because its pathogenesis involves multiple inflammatory/proinflammatory factors, and it is characterized by chronic low-grade inflammatory disease [86]. The model of early diabetic nephropathy was established and showed increases in serum microalbuminuria, NADPH oxidase expression, and PKR-like eukaryotic initiation factor 2α kinase (PERK) level, and a decrease in connexin 43 (Cx43) in renal tissue. Upregulation of nuclear transcription factors, such as PERK, reveals the presence of endoplasmic reticulum stress (ER stress) [87]. Cx43, a family of connexins, may play a key role in exchanging small molecules within glomeruli and tubules in the kidney, which is essential for normal renal function [43]. Argirein, a derivative of rhein, can be produced by combining rhein with L-arginine by forming a hydrogen bond. It has anti-inflammatory activity derived from rhein. Argirein (200, 100, and 50 mg/kg) is more effective than aminoguanidine (AMG) (100 mg/kg), which has anti-inflammatory activity as a positive reference agent in reversing the above changes [43].

A strong inflammatory response is further stimulated by the formation of uric acid crystals in the kidneys [88]. Anti-hyperuricemia and anti-inflammatory effects can reduce kidney damage. There are few drugs available to treat hyperuricemia and kidney disease, such as uricosuric agents and xanthine oxidase inhibitors (XOD) [89]. However, when renal injury has already occurred, uricosuric drugs may be less effective. On the other hand, allopurinol, one of the most commonly used drugs, is prone to severe allergy and agranulocytosis and aggravates nephrotoxicity by damaging pyrimidine metabolism [90]. Therefore, for a new compound is required that can reduce uric acid and treat kidney disease with relatively small side effects. In mice, adenine and ethambutol were used to establish an experimental model of hyperuricemia and nephropathy [58]. In hypouricemic mice, rhein effectively decreased serum uric acid (Sur), serum creatinine (Scr), and blood urea nitrogen (BUN), and inhibited XOD activity [58]. On the other hand, allopurinol could significantly lower Sur and Scr levels and attenuate XOD activity, but not reduce BUN levels effectively [58]. At the same time, important renal inflammatory factors prostaglandin E2 (PGE_2_), tumor necrosis factor-α (TNF-α) and interleukin-1β (IL-1β) were also measured. Rhein decreased the levels of PGE_2_, TNF-α, and IL-1β in the kidneys of model mice in a dose-dependent manner [58]. Compared with allopurinol, a high dose of rhein (300 mg/kg) had a stronger inhibitory effect on these factors [58]. Rhein lysinate (RHL) is the lysine salt of rhein, and its protective effects in a mouse model of DN were also studied [65]. With the treatment of RHL, significant reductions in TNF-α, IL-6, and NF-κB expression and phosphorylation were observed in the kidneys [65]. There are other studies that reveal the mechanism of rhein action in uric acid nephropathy (UAN). The abnormal expression of long noncoding RNAs (lncRNAs) is related to the biological processes of a variety of diseases such as cell growth and differentiation, development, and inflammation [91]. ANRIL is a well-known functional lncRNA that plays a key role in a variety of human diseases [92]. It has been shown that treatment of rhein and allopurinol in UAN rats significantly decreased TNF-α, IL-1β, IL-6, and IL-8 levels, and effectively suppressed the expression of ANRIL [19]. In TNF-α-induced NRK-52E cells, rhein at doses of 20 and 40 μg/mL significantly inhibited the expressions of IL-6, IL-8, and ANRIL [19]. However, this effect of rhein was abolished through upregulating the expression of ANRIL. Rhein played a protective role in urate nephropathy by alleviating the lncRNA ANRIL-mediated inflammation response [19]. Other studies have indicated that lncRNA-cytochrome oxidase subunit 2 (Cox2) regulates SWI/SNF-mediated chromatin remodeling and promotes macrophage-mediated late inflammation [93,94]. Hu J et al. (2020) discovered that lincRNA-Cox2 was involved in the progression of UAN via regulating the inflammatory response [72]. Mir-150-5p is associated with the regulation and pathogenesis of immune diseases. With the help of predictive analytics software, they found that lincRNA-Cox2 interacted with miR-150-5p and revealed that STAT1 was a target gene of miR-150-5p. It was found that rhein attenuated renal inflammatory injury via lincRNA-Cox2/miR-150-5p/STAT1 [72].

The mortality rate of AKI is about 70–80 % [95,96]. Currently, renal replacement therapy (RRT) is the only effective treatment for AKI [97]. Inflammatory response inherent to sepsis is thought to be the direct mechanism of AKI [98]. Based on rhein’s anti-inflammatory pharmacological activity, Yu C et al. (2015) explored the effects of rhein on sepsis-induced AKI by using lipopolysaccharide (LPS) and cecal ligation and puncture (CLP) models [55]. The potential mechanism of rhein may be related to its anti-inflammatory effects. Rhein inhibited the activation of NF-κB via restraining the expression and phosphorylation of the relevant proteins in the NF-κB signal pathway and hindering the transcription of NF-κB p65 [55]. It brings a new research direction for solving AKI related to endotoxemia. At the same time, the research of Liu M et al. (2021) also confirms this point [73]. The 5/6 nephrectomy model (5/6 Nx) in rats and LPS-induced HK-2 cells were used in this study, and the results indicated that rhein inhibits inflammatory signaling pathways via decreasing the production of TNF-α, IL-6, and monocyte chemotactic protein (MCP-1) [73]. In addition, by inhibiting NF-κB phosphorylation, rhein diminished LPS-induced NF-κB activation [73]. The above results clearly indicate that rhein can be a promising therapeutic agent for renal disease by inhibiting multiple inflammatory mediators [55,73].

Glomerulonephritis (GN) is an inflammatory process in the glomerular capillaries accompanied by acute nephrotic syndrome, especially hematuria and proteinuria [99]. The most common form of GN is chronic glomerulonephritis (CGN) [100] with signs and symptoms of hematuria, proteinuria, and renal impairment [101]. Chen Q et al. (2022) established a rat model of CGN and explored the mechanism of the kidney protection effects of rhein on CGN [76]. TNF-α, IL-1β, IL-6, and ICAM-1 levels were lower in the rhein group than in the CGN group in rat kidney tissue. The experimental results showed that the rhein could inhibit the inflammatory reaction of CGN [76]. In the rhein groups, the expression of Nucl-NF-κB was significantly decreased, while the expression of Cyto-NF-κB was significantly increased. From what has been discussed above, this showed that rhein could be a potential drug for the treatment of CGN via inhibiting the NF-κB signaling pathway [76]. NF-κB activation and Nrf2 impairment were the most significant pro-inflammatory and anti-inflammatory signals. Luo L et al. (2021) revealed that a Shenkang injection (SKI) and its components including chrysophanol, emodin, and rhein [102] could inhibit oxidative stress and inflammation by regulating the IκB/NF-κB and Keap1/Nrf2 signaling pathways [75].

One of the most commonly diagnosed types of GN in adults and children is immunoglobulin A nephropathy (IgAN) [103]. In IgAN, toll-like receptor 4 (TLR4) induces pro-inflammatory cytokines that cause mesangial cell injury. Blocking TLR4-mediated inflammatory and profibrotic signaling is a novel strategy for treating IgAN [104]. The renal anti-aging protein Klotho has strong anti-inflammatory and nephroprotective properties [105]. Klotho recovery has become an attractive treatment strategy for renal inflammatory diseases. Klotho inhibits TLR4 through deglycosylation and thereby negatively controls the TLR4-related inflammatory signaling pathways [106]. Rhein inhibited TLR4 by maintaining the expression of Klotho, which has anti-inflammatory and nephroprotective properties [67]. Chen X et al. (2015) showed that rhein alleviated kidney damage by regulating the expression of TLR4 and TLR9 in the renal tissue of IgAN rats [53].

### 2.3. Antioxidant Proprieties of Rhein

Kidney function and morphology change significantly with age, while renal failure susceptibility increases [107]. During aging, oxidative stress leads to kidney damage. The SAMP10 mouse is an ideal model for studying glomerulonephritis and senescence [108]. By reducing malondialdehyde (MDA) levels and increasing superoxide dismutase (SOD) and glutathione peroxidase (GSH-Px) levels, the median survival time of SAMP10 mice was increased by RHL [48]. One of the main causes of AKI is drug-induced nephrotoxicity. Acetaminophen (APAP) can cause fulminant liver necrosis and nephrotoxicity when used in high doses [109]. Many chemicals have been proposed to induce nephrotoxicity via the depletion of GSH and lipid peroxidation [110]. Excessive production of free radicals caused by oxidative stress can directly damage the cell membranes of hepatocytes and kidneys through lipid peroxidation or other ways. This is followed by a series of cellular events, such as a massive release of inflammatory mediators or cytokines, which eventually cause liver and kidney damage [111]. Based on the antioxidant activity of rhein, Zhao Y et al. (2011) clarified the protective effects of rhein on liver and kidney injury induced by acetaminophen. Rhein attenuated APAP-induced hepatotoxicity and nephrotoxicity in a dose-dependent manner [23]. The levels of serum glutamate-pyruvate transaminase (GPT), glutamate-oxaloacetic transaminase (GOT), urea nitrogen (UREA), creatinine (Crea), and reactive oxygen species (ROS) production were significantly decreased, and the contents of nitric oxide (NO), MDA, and GSH were recovered in the rhein treatment group [23]. Rhein relieved APAP-induced liver and kidney injury by ameliorating oxidative stress [23]. Diacerein (DIA) is used to treat osteoarthritis. DIA enters the body and is rapidly converted into its active metabolite rhein [63]. Furthermore, studies have been conducted one after another on whether DIA can alleviate AKI. The antioxidant effects of DIA were reported to protect renal function against doxorubicin-induced AKI [63]. Crea, MDA, NOx, GSH, histopathological, and immunohistochemical parameters were improved in the DIA group [63]. The antioxidative effect of DIA in alleviating renal injury was also demonstrated in a model of AKI induced by glycerol [69]. Glycerol treatment resulted in an increase in the renal MDA level and a decrease in catalase activity and finally caused DNA damage. However, DIA attenuated glycerol-induced renal damage in a concentration-dependent manner, significantly reduced renal oxidative damage, and decreased the expressions of RIPK3, MLKL, Bax, TNF-α, and HO-1 in the kidneys [69]. The production and clearance of ROS and regulation of fibrosis are closely related to the SIRT3/FOXO3a signaling pathway that can regulate mitochondrial oxidative and metabolism [112]. ROS production was increased in 5/6 nephrectomied (5/6Nx) rats, which is an animal model of progressive CKD. After treatment with rhein, the ROS level was significantly reduced [71]. In HK-2 cell assays, the antioxidant and antifibrotic effects of rhein were abrogated by SIRT3 knockdown. By activating the SIRT3/FOXO3a signaling pathway, rhein may alleviate oxidative stress and reduce interstitial fibrosis [71].

### 2.4. Antifibrotic Proprieties of Rhein

CKD and chronic kidney failure (CRF) develop as a result of renal fibrosis. The pathological mechanism of renal fibrosis is relatively complex. There is a variety of stimulating factors or mediators, such as growth factors, cytokines and toxins, which induce the occurrence of fibrosis through a variety of mechanisms and signal pathways [113,114]. TGF-β has been proven to be a major pathogenic factor for the progressive development of renal fibrosis [115]. TGF-β can induce renal tubular epithelial cells to transform into renal mesenchymal fibroblasts through the epithelial–mesenchymal transition (EMT) process [116]. In addition to the TGF-β pathway, the Notch, Wnt, and Hedgehog signaling pathways can also be activated in response to renal injury, thereby promoting renal fibrosis [117]. Currently, many scholars have carried out studies of rhein against renal fibrosis. The therapeutic effect of rhein on renal interstitial fibrosis caused by unilateral ureteral obstruction (UUO) was studied by He D et al. (2011) [44]. Rhein treatment significantly improved renal interstitial fibrosis by inhibiting the expressions of α-smooth muscle actin (α-SMA), TGF-β1 and its type I receptor, and FN deposition [44]. Rhein inhibited the phenotypic transformation of rat renal interstitial fibroblasts induced by TGF-β1 [44]. Rhein also exerted anti-fibrotic effects by reducing FN and α-SMA expressions in a rat model of IgAN [49].

TGF-β regulates renal fibrosis progression through classic and non-classic pathways. The classical TGF-β pathway includes two signaling pathways, namely TGF-β/Smad and bone morphogenetic protein (BMP) [115]. However, they have opposite effects although they have similar downstream Smad signaling pathways. Smad3 is a key downstream mediator of TGF-β signal transduction [115]. BMP7 is a member of the TGF-β superfamily that antagonizes the effects of TGF-β [118]. Like liver growth factor (HGF), they both have anti-fibrotic effects [118]. Combined therapy of rhein and Danshensu (DSS) had a certain renal protective effect on chronic kidney damage. The mechanism may be related to anti-inflammatory and anti-fibrosis by downregulating the NF-κB-related pathway and inhibiting the TGF-β/Smad3 pathway, respectively [56]. Other studies have shown that rhein improved renal function and reduced renal fibrosis and interstitial inflammation by inducing HGF and BMP7 production [50].

In addition to the classical Smad signaling pathway, TGF-β can also regulate the downstream cellular response through other non-classical pathways and then adjust the pathological process of renal fibrosis [115]. p38 mitogen-activated protein kinase (MAPK) is one of the atypical signaling pathways of TGF-β1. TGF-β1 activates the downstream signaling pathway MKK3-p38 MAPK cascade through the activation of TAK1, ultimately leading to cell fibrosis [119]. Rhubarb and Astragalus capsules (RAC) that contain 2.25 mg/g rhein have been used in a clinical treatment for chronic kidney disease [70]. Zeng X et al. (2020) showed that RAC had an antifibrotic effect on UUO-induced renal interstitial fibrosis in a rat model, possibly due to inhibiting apoptosis by regulating the TGF-β1/p38 MAPK pathway [70]. c-JunNH2-terminal kinase (JNK) signaling plays a pathogenic role in renal tubular cell apoptosis and fibrosis [120]. Rhubarb, a major herb of Dahuang Fuzi Decoction (DFD), has been reported to attenuate TGF-β1-mediated hepatic stellate cell migration by interfering with JNK phosphorylation [121]. Furthermore, it was found that the TGF-β1-JNK pathway was activated in adenine-induced renal injury rats, which then activated the Bcl-2/Bax-caspase-3 mitochondrial pathway and finally induced apoptosis [52]. By inhibiting the activation of the TGF-β1-JNK signaling pathway, DFD reduced adenine-induced renal injury and apoptosis of renal tubular epithelium cells [52]. It is known that severe apoptosis of renal tubular epithelial cells can cause renal tubular atrophy and interstitial fibrosis, while inhibiting the apoptosis of renal tubular epithelial cells can delay renal interstitial fibrosis [122]. Consistent with the study performed by Lian Y et al. (2014) [51], astragalus polysaccharide improved renal function, reduced cell apoptosis, and mitigated endoplasmic reticulum stress (ERS) in combination with rhein. Chen Y et al. (2019) also found that rhein alleviated UUO-induced renal failure by inhibiting the apoptosis of renal tubular cells and had a negative regulatory effect on renal interstitial failure [21]. Integrin-linked kinase (ILK) is a downstream factor of TGF-β1. ILK is involved in signal transduction pathways such as integrin, TGF-β1/Smad and MAPK, and has been shown to regulate cell adhesion, migration, and extracellular matrix accumulation [123]. Matrix metalloproteinase-9 (MMP-9) inhibits the degradation of the extracellular matrix and promotes its accumulation and thereby promotes renal interstitial fibrosis [124]. MMP-9 and its tissue inhibitor TIMP-1 are normally used in homeostasis, and the MMP-9/TIMP-1 ratio fluctuates around 1 [125]. The ratio of MMP-9/TIMP-1 is considered as a potential indicator for evaluating renal fibrosis. It has been reported that ILK can enhance the activity of MMP-9 promoter [126] and imbalance the MMP-9/TIMP-1 ratio. It had been found that ILK was overexpressed, and the MMP-9/TIMP-1 ratio was disturbed in diabetic nephropathy EMT. In HK-2 cells, rhein inhibited high-glucose-induced EMT by inhibiting ILK expression and regulating the MMP-9/TIMP-1 ratio to relieve renal fibrosis [46]. Peroxisome proliferator-activated receptor-α (PPARα) is strongly expressed in renal tubular epithelial cells (TECs) and is involved in lipid metabolism [127]. The accumulated evidence has shown that TGF-1 produced abnormalities in the fatty acid oxidation (FAO) pathway in renal epithelial cells, decreased the expression of PPAR and its downstream proteins, and accelerated the onset of renal fibrosis. Nevertheless, rhein was able to prevent renal fibrosis by activating the PPARα–CPT1A axis [77]. Rhein attenuated autophagy and renal fibrosis in rats with renal tubular injury induced by adenine [64]. In addition, rhein inhibited autophagy by regulating key molecules in the adenosine monophosphate-activated protein kinase (AMPK)-dependent rapamycin (mTOR) signaling pathway, as well as the extracellular signal-regulated kinase (Erk) and p38MAPKs signaling pathways [64].

Klotho is an anti-aging gene discovered in 1997. With the deepening of the understanding of its biology, it has been found that its functions have gone beyond the category of “anti-aging”, such as antioxidant, anti-inflammatory, regulation of calcium and phosphorus metabolism, inhibition of vascular calcification, inhibition of apoptosis and tissue and organ fibrosis, etc., and the inhibitory effect of Klotho on fibrosis is especially worthy of attention [128,129]. In some studies, Klotho expression in renal tissue is negatively correlated with renal interstitial fibrosis (RIF) [130] and glomerular sclerosis [131]. Administration of exogenous soluble Klotho reduced the degree of RIF and improved renal function, which confirms the inhibitory effect of Klotho on RIF [131]. Physiological modifications, such as methylation of DNA and post-translational modifications of histones, can also affect renal fibrosis [132]. Abnormal expression of DNA methyltransferases (DNMT), hypermethylation of Klotho promoter and Klotho inhibition are associated with UUO induced renal fibrosis. Rhein, a Klotho upregulator, significantly reversed Klotho downregulation in UUO-induced fibrotic kidneys [61]. Zhang Q et al. (2017) also discovered that rhein reversed the loss of Klotho by inhibiting aberrant DNMT expression and hypermethylation of the Klotho promoter in an adenine animal model [62].

### 2.5. Benefits of Rhein Via Drug-Transporter

Renal transporters transport endogenous substances, poisons, and drugs from the blood to the urine. As a result of renal injury, uptake transporters and efflux transporters are altered, which affects toxic excretion and aggravates renal injury [133]. Previous research by our group has shown that the expressions of organic anion transporter 1 (OAT1), OAT3, and multidrug resistance related protein 2 (MRP2) were significantly decreased after cisplatin-induced AKI, which reduced the excretion of endotoxin and aggravated renal injury [133]. There are few studies on the relationship between the various pharmacological effects of rhein and transporters. Zhu Y et al. (2022) [24] showed that the gene levels and protein expressions of the renal transporters including Oat1, Oat3, Organic cation transporter 2 (OCT2), mammal multidrug, and toxin extrusion proteins 1 (Mate 1), Mrp2, and P-glycoprotein (P-gp) in vancomycin-induced nephrotoxicity (VIN) were significantly decreased. Plasma creatinine, BUN, and plasma indoxyl sulfate were not excreted efficiently. Rhein reversed the expressions of the above transporters, and thereby promoted the excretion of endotoxins and finally alleviated renal injury [24]. The discovery of VIN’s pathogenesis expands the field of study on the kidney protection effects of VIN. In addition, it provides a new mechanism for rhein to alleviate drug-induced AKI [24].

## 3. Toxicological Effects in Kidney

**Figure 2:**
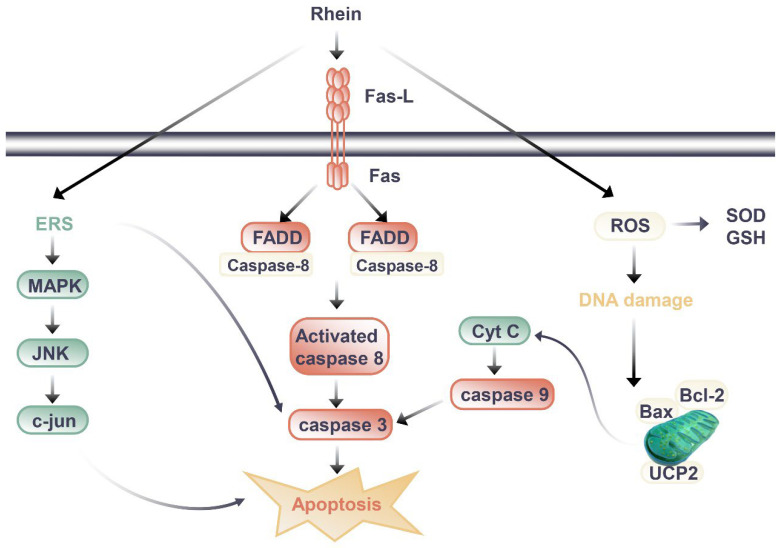
Signal pathway of nephrotoxic effect of rhein.

Total rhubarb anthraquinones (TRAs) include emodin, rhein, chrysophanol, aloe emodin, and other substances [134]. However, the clinical cases of liver injury and the progression of kidney disease caused by TCM contained TRAs are increasing. Rhein is an important ingredient in TRAs, which affects the kidneys in a positive and negative way. Studies on the nephrotoxicity effects of rhein and the related mechanisms are shown in Table 2 and Figure 2.

### 3.1. Rhein Nephrotoxicity: Mechanisms of Action and Possible Causes

Through the literature review, the difference between the kidney protection and nephrotoxicity effects of rhein may be related to the dosage and duration of rhein. When rhein exerted kidney protection effects, the dosage of rhein in animal experiments was mostly 20 to 150 mg/kg/day, and the duration of administration was mostly less than 14 days, with the longest time being 8 weeks when the rhein was at a slightly lower dosage. In mice, HU Y et al. (2019) observed renal toxicity after long-term administration of rhein [139]. Mice were randomly divided into three groups: blank group, low-dose rhein group (0.175 g/kg), and high-dose rhein group (0.35 g/kg). The drug was administered by gavage for 60 days [139]. Compared to the blank group of the same sex, BUN and SCr levels of the mice in the administration group were increased, and the body weight of the mice in the rhein high-dose group decreased [139]. The renal index of male mice in the administration group decreased significantly, the content of GSH-Px decreased, and the expression of TGF-β1 increased in the male mice in the rhein high-dose group [139]. Its potential toxic mechanism may be caused by the imbalance of the glutathione antioxidant system that can induce excessive oxidation, inflammatory reaction, and apoptosis induced by the activation of caspase-3 [139].

In cell experiments, the transition between kidney protection and nephrotoxicity is more closely related to the dose and duration of administration. Da H et al. (2009) evaluated the cytotoxic effects of emodin and rhein in HK-2 cells [135]. The results showed that both emodin and rhein could inhibit the growth of HK-2 cells, but the inhibitory effect of rhein was weaker than that of emodin [135]. From the experimental results of rhein on the survival rate of HK-2 cells, we found that rhein had an obvious inhibitory effect on cell proliferation when they were treated with 40 μM for 24 h, and the inhibitory effect gradually increased with the extension of incubation time, while cell proliferation could be significantly inhibited after 12 h of administration when the dosage of rhein was 100 μM [135]. It was preliminarily elucidated that rhein caused renal injury by inducing apoptosis.

Researchers have also carried out a series of studies to further clarify the nephrotoxicity mechanisms of rhein. Most studies have shown that it is related to the induction of apoptosis (Figure 2). In addition to death-receptor signaling, the mitochondrial death pathway, oxidative stress, and endoplasmic reticulum stress all contribute to apoptosis [140]. It was found that rhein directly inhibited HK-2 cell growth and increased apoptosis in a dose- and time-dependent manner according to the study by Yang J et al. (2015) [136]. Rhein (50 and 100 μM, 24 h after administration) increased the mRNA levels of amino terminal kinase (c-Jun), activated transcription factor-2 (ATF-2), and caspase-3, and upregulated the expression of p38 MAPK and cleaved caspase-3. These results suggest that rhein may induce apoptosis in HK-2 cells through the MAPK signaling pathway [136]. Hao S et al. (2015) [137] also found rhein could dose-dependently inhibit the viability of HK-2 cells, increase the release of lactate dehydrogenase (LDH) and apoptosis rate, and significantly upregulate the mRNA or protein expressions of Fas, FasL, FADD, caspase-3, caspase-8, and Cytochrome C (Cyt-c). Apoptosis induced by the Fas pathway may be the mechanism behind rhein’s toxic effects on HK-2 cells in vitro. In another study [138], rhein reduced mitochondrial membrane potential and intracellular ATP level, released Cyt-c, and decreased Bcl-2 and Bax protein levels in HK-2 cells. Meanwhile, rhein increased the intracellular ROS level and inhibited mitochondrial uncoupling protein 2 (UCP2) expression, which regulates mitochondrial membrane potential, ROS generation, and ATP synthesis [138]. Rhein inhibited the expression of UCP2, significantly enhanced oxidative stress in cells, and thus promoted cell apoptosis, indicating the potential role of UCP2 in rhein nephrotoxicity [138].

### 3.2. Methods for Controlling Rhein Toxicity

In order to use rhein reasonably and safely, there may be some measures we should take. On the one hand, it is important to control the dosage and duration of rhein administration as described above; on the other hand, the compatibility of TCM can enhance its protective effects and reduce toxicity. For instance, different doses of astragaloside IV (10, 20, and 40 μM) could reduce the occurrence of rhein-induced vacuolation, cell fusion, and the increase in necrotic cells in HK-2 cells [141]. After the combination of rhein and astragaloside IV in HK-2 cells for 48 h, the cell inhibition rate and LDH leakage rate were significantly reduced [141]. The compatibility significantly increased the contents of SOD and GSH in cells and downregulated the expression of MDA, which indicates that astragaloside IV could significantly inhibit the oxidative stress injury caused by rhein and subsequently protect cells [141].

## 4. Conclusions and Future Prospects

This review comprehensively summarized the research progress and molecular targets of rhein in terms of its positive and negative regulatory effects on the kidneys. In most cases, rhein has been shown to have a protective effect on the kidneys. At present, there are limited studies on the nephrotoxicity of rhein, and most of them are in vitro cell experiments. However, the data still shows that the difference between the kidney protection and nephrotoxicity of rhein may be related to the dosage and duration of rhein administration. More in vitro and in vivo experiments on the nephrotoxicity of rhein are needed for further investigation. This review summarized all the existing literature and the specific mechanisms on the positive and negative regulatory effects of rhein on the kidneys, which is convenient for basic researchers to better and more quickly refer to relevant references and provide convenience for further research. It is hoped that the nephroprotective effects of rhein can be fully exerted and its nephrotoxicity can be avoided through the joint efforts of researchers.

## Figures and Tables

**Table 1:** Nephroprotective effects of rhein, its derivatives, and its mechanisms of action.

Nephrotoxicity, Dose, Treatment Schedule	Animal Model/ Tissue Used	Rhein or Derivatives Dose, Treatment Schedule	Effector Mechanisms	References
db/db mice were designated as diabetic on the basis of appearance of obesity	C57BL/KsJ db/db and db/m mice	Rhein: 150 mg/kg/day by oral gavage for 12 consecutive weeks	Reduced urinary albumin excretion, decreased body weight, plasma glucose, cholesterol, triglyceride, and low-density lipoprotein, relieved glomerular hypertrophy, mesangial expansion, and proliferation, and decreased the expression of FN and TGF-β1	[40]
MCGT1 and MCLacZ cells: 8 mM glucose and 20% newborn calf serum	Normal rat MCGT1 cells, MCLacZ cells, and mesangial cells	Rhein: 25 μg/mL and 50 μg/mL for 48 h	Inhibited the increased activity in the hexosamine pathway, inhibited hypertrophy of MCGT1 cells, inhibited FN synthesis, partly reduced glucose uptake, high glucose-induced GFAT activity	[41]
C57BL/KsJ db/db mice	C57BL/KsJ db/db and db/m mice	Rhein: 150 mg/kg/day by oral gavage for 12 consecutive weeks	Regulated dyslipidemia, reduced TGF-β and FN expression	[42]
Single injection of STZ 65 mg/kg, ip	Rats	Argirein (a derivative of rhein): 200, 100, and 50 mg/kg/d, po	Suppressed inflammatory cytokines	[43]
UUO operations, recombinant human TGF-β1 at a final concentration of 2 ng/mL	Male CD-1 mice and NRK-49F cells	Rhein: 150 mg/kg/day by orally administrated for 3 and 7 days. Rhein: a final concentration of 0.01, 0.1, and 1 ng/mL for 48 h	Attenuated deposition of FN, suppressed the expression of TGF-β1 and its type I receptor in kidneys obstructed by blockage	[44]
Acetaminophen (APAP) 2.5 g/kg/d by intragastrically once	Male SD rats	Rhein: 40, 20, and 10 mg/kg/day	Oxidative stress	[23]
db/db mice	db/db mice	Rhein: 120 mg/kg/day for 8 weeks	Improved glucose tolerance by improving insulin secretion during the first phase and early phase. improvement of β cells function	[45]
30 mM D-glucose + 10% FBS for a further 48 h	HK-2 cells	Rhein: 25, 50, and 100 µg/mL for a further 48 h	Inhibited ILK and regulated abnormal MMP-9/TIMP-1 ratio in EMT	[46]
Polybrene (8 mg/mL) was added to the cells along with lentivirus stock and incubated for 6 h	db/db mice and NIT-1 cells	Rhein: 120 mg/kg/day by oral gavage for 8 weeks. Rhein: 1 mg/mL for 5 min (in NIT-1 cells)	Stabilized mitochondrial morphology in pancreatic β cells to prevent hyperglycemia-induced cell apoptosis	[47]
SAM model	Male SAMP10 and SAMR1 mice	RHL: 25 mg/kg/day and 50 mg/kg/day by orally administration until 50% of the mice in each group died	Oxidative stress had powerful immune-modulating effects and reduced lipofuscin deposition in the kidneys, reduced the production of inflammatory factors, inhibited the immune response by blocking the TNF-α/NF-κB signaling pathway, blocked the progression of renal interstitial fibrosis	[48]
IgAN experimental animal models were established with BSA-LPS-CCL4	Female SD rats	Rhein: 100 mg/kg/day from the 7th week until the 10th week. Rhein: 100 mg/kg/day from the 1st week until the 10th week	Inhibited the expression of FN and α-SMA	[49]
CAN model	Inbred male rats	Rhein: 100 mg/kg/day by gavage for 16 weeks	Produced HGF and BMP7 to reduce fibrosis and inflammation in renal tissues	[50]
Adenine (2%) suspension: 200 mg/kg/d by oral gavage for 28 days, during the subsequent 3 weeks. The model group, low-dose APS plus rhein, and high-dose APS plus rhein groups received adenine by gavage every other day	Male Wistar rats	APS (200 or 400 mg/kg) plus rhein (25 or 100 mg/kg) combination by gavage for 7 weeks	Inhibited apoptosis	[51]
Adenine (2%) at a dose of 150 mg/kg for 2 weeks, from the 3rd week, the rats in the Vehicle, DFD, and AP groups were given 150 mg/kg of 2% adenine every 3 days to avoid a rapid recovery of renal function	Rats	DFD: 2.5 g/kg/day by gastric gavage from the 3rd week to the 5th week (3 weeks in total)	Suppressed TGF-β1-JNK pathway activation	[52]
IgAN experimental animal models were established with BSA-LPS-CCL4	Male SD rats	Rhein: 100 mg/kg from the 7th week up to 10th week. Rhein: 100 mg/kg/day from the 1st week to the 6th week	Inhibited TLR4-mediated profibrotic signals and expression of the profibrotic molecule TGF-β1	[53]
Intraperitoneal injection of 10 mg/kg of LPS once at the 7th day. The CLP procedure [54]	BALB/c mice	Rhein: 20, 40, and 80 mg/kg/day by oral administration for 7 days	Inhibited NF-κB activation and had anti-inflammatory and immunomodulatory properties	[55]
5/6 subtotal nephrectomy. Cells underwent serum starvation for 24 h before treatments with 5 ng/mL TGF-β	SD rats and HK-2 cells	Rhein: 100 mg/kg/day by oral administration for 12 weeks. Rhein: 30 μM in cells	An inhibitory effect on EMT and downregulation of the TGF-β/Smad3 pathway	[56]
Type 2 diabetic rats: a high-glucose and high-fat diet combined with STZ (35 mg/kg body mass)	Male SD rats	Rhein: 50, 100, and 150 mg/kg/day for 16 weeks	Reduced kidney damage in diabetic rats by boosting SIRT1 expression, reducing insulin resistance, and decreasing dyslipidemia	[57]
Adenine (150 mg/kg) and ethambutol (250 mg/kg) were administration by intragastric administration for 14 days	Mice of the Kun-Ming strain	Rhein: 75, 150, and 300 mg/kg/day for 14 days	Suppressed the expression of TGF-β1 and reducing the synthesis of proinflammatory cytokines such as IL-1, prostaglandin E2, and TNF	[58]
db/db mice. Podocyte: HG: 30 mM of glucose for 48 h	Male db/db and db/m mice, Podocyte	Rhein: 120 mg/kg/day by oral administration for 12 weeks. Rhein: 25 μg/mL for 48 h (cells)	Regulated the expressions of the Wnt/β-catenin pathway, GSK3β, nephrin, and PPAR-γ	[59]
A mouse UUO model was established as described previously [60]. HEK293 cells were transfected with a control plasmid (containing scrambled sequences) or with a Klotho-specific shRNA plasmid, and then treated with TGF-β (5 ng/mL)	C57BL/6 male mice and HEK293 cells	Rhein: 120mg/kg/day by oral gavage once before UUO operation or 3 days after UUO surgery. Rhein: 10 μg/mL for 1 h in the presence or absence of TGF-β for additional 48 h	Reversal of Klotho loss by interrupting TGF-β/Smad and Wnt/β-catenin signaling, demethylates by modulating DNMTs	[61]
Adenine mouse model: 0.2% adenine-containing diet for 8 weeks. HK2 cells: Klotho interference plasmids first	C57BL/6 male mice and HK2 cells	Rhein: 120 mg/kg/day orally for 8 weeks. Rhein: 10 μg/mL for 48 h	Reversed renal Klotho deficiency	[62]
Rats: Dox (15 mg/kg) by i.p. at day 11	Adult male Wistar rats	Diacerein: 25 and 50 mg/kg/day orally for 15 days	Antioxidant and anti-inflammatory activities	[63]
2% Ade: 150mg/kg for 2 weeks; from the 3rd week to the 5th week, every 3 days, 2% Ade was given. NRK-52E cells transiently transfected with pmRFP-LC3, or Deptor	Male SD rats and NRK-52E cells	Rhubarb: 1 g/kg/day by gastric gavage for 3 weeks (from the 3rd week to the 5th week). Rhein: 1, 5, and 10 μg/mL for 1 h in NRK-52E cells; 5 μg/mL for 1 or 6 h in NRK-52E cells	Inhibition of autophagy by AMPK/mTOR, p38/Erk MAPKs, and Akt-independent signaling pathways	[64]
KK/HlJ mice: STZ (50 mg/kg/day) by intraperitoneal injection for 5 consecutive days	Male C57BL/J mice and KK/HlJ mice	RHL: 25 and 50 mg/kg/day for 15 weeks	Decreased kidney inflammation by reducing oxygen free radical levels, blocking the TNF-α/NF-κB biochemical pathway, and improving renal function	[65]
LPS-induced AKI was adopted from a previous study [66], LPS intraperitoneal injection (10 mg/kg). LPS (100 ng/mL for RAW or 300 ng/mL for HEK293) for 12 h.	C57BL/6 male mice, RAW cells, THP-1 cells, HK2 cells, and HEK293 cells	Rhein: 120 mg/kg/day by oral gavage. Rhein: 5 or 10 μg/mL for 12 h (RAW cells); 10 μg/mL for 12 h (HK2 cells); 10 μg/mL for 30 min (HEK293 cells)	Corrected the inverted changes in Klotho and TLR4 and reduced the downstream inflammatory response of TLR4	[67]
UUO was induced through ureteral ligation	Male SD rats	Rhein: 150 mg/kg/day by oral gavage for 7 days	Suppressed the apoptosis of tubular cells	[21]
Adenine-induced hyperuricemia in rats [68]. NRK-52E cells: TNF-α (25 ng/mL)/IL-1β (10 ng/mL) (Peprotech) for 24 h	Male SD rats and NRK-52E cells	Rhein: 75, 150, and 300 mg/kg/d underwent gastric perfusion for 14 days. Rhein: 10, 20, and 40 μg/mL for 2 h (cells)	Anti-inflammatory and kidney protection	[19]
Rats: prevented from drinking water 6 days, then they received glycerol (10 mL/kg, 50% *v*/*v* in sterile saline) in each hind limb muscle	Male Wistar albino rats	Diacerein: 25 and 50 mg/kg/day by orally administrated for 7 days	Modulated oxidative stress, inflammation, apoptosis, and necroptosis	[69]
UUO rats	Male SD rats	RAC: 81.46, 162.93 and 325.86 mg/kg after 7 days post-surgery, with a duration of 7 days or 14 days	Anti-fibrotic potency due to the inhibition of renal tubular epithelial cells from apoptosis through regulating the TGF-β1/p38 MAPK pathway	[70]
The 5/6Nx operation. HK-2 cells: H_2_O_2_ (2 mM) for 4 h	Male SD rats and HK-2 cells	Rhein: 50, 100, and 150 mg/kg/day for 1 month	Activated the SIRT3/FOXO3a signaling pathway	[71]
TCMK-1 cells: 200 μM uric acid	TCMK-1	Rhein: 10, 20 and 40 μg/mL	Anti-inflammatory effects through downregulation of lincRNA-Cox2	[72]
The 5/6Nx operation. HK-2 cells: Rhein (25 or 50 μg/mL) for exposing to LPS (0.1, 1, 2 μg/mL) for 6 h, 12 h, or 24 h	Male SD rats and HK-2 cells	Rhein: 25 and 50 μg/mL/day via tail vein injection for one week after surgery to 30 days after surgery. Rhein: 25 or 50 μg/mL for 6 h, 12 h or 24 h	Reduced inflammation via NF-κB signaling	[73]
Adenine (200 mg/kg/d) for 3 weeks [74]	Male SD rats	Rhein: 150 mg/kg/day orally for 3 weeks	Ameliorated renal fibrosis by regulating the IκB/NF-κB and Keap1/Nrf2 Signaling Pathways	[75]
VCM-induced nephrotoxicity: i.p. administration of VCM (400 mg/kg) for 7 days. NRK-52E cells: VCM (4 mM) for 24 h	Male Wistar rats NRK-52E cells	Rhein: 12.5, 25, and 50 mg/kg/day for 10 days. Rhein: 0.6, 3, and 15 μM for 2.5 h before challenging with VCM (4 mM) for 24 h	Regulated the Nrf2 signaling pathway	[24]
CGN: C-BSA 1ml (2.5 mg/mL) for 6 weeks by injection	Male SD rats	Rhein: 100 mg/kg/day orally for 6 weeks	Inhibited the NF-κB signaling pathway	[76]
Rats: uIRI surgery. NRK-52E and HK-2 cells: TGF-β1 (10 ng/mL)	SD rats, HK-2 cells and NRK-52E cells	Rhein: 50, 80, and 120 mg/kg/day orally from days 0 to 13 days after surgery. Rhein:0.25, 0.5, and 1 μg/mL for 48 h	Played an antifibrotic role via regulating the PPAR–α–CPT1A–l-palmitoyl–carnitine axis	[77]

**Table 2:** Nephrotoxic effect of rhein and the related mechanisms.

Animal Model/Tissue Used	Rhein or Derivatives Dose, Treatment Schedule	Effector Mechanisms	References
HK-2 cells	Rhein: 10, 20, 40, 80, and 100 μM for 12, 24, and 48 h	Induced apoptosis	[135]
HK-2 cells	Rhein: 0, 25, 50, and 100 μM for 12, 24, and 48 h	Induced apoptosis by the MAPK signal transduction pathway	[136]
HK-2 cells	Rhein: 10, 25, 50, 100, and 200 μM for 12, 24, and 48 h	Induced apoptosis by the Fas-dependent pathway	[137]
HK-2 cells	Rhein: 0, 25, 50, and 100 μM for 12, 24, and 48 h	Induced apoptosis by the UCP2-related mitochondrial pathway	[138]
Kunming mice	Rhein: 0.175 and 0.35 g/kg/day by oral gavage for 60 days	Caused an imbalance in the glutathione antioxidant system, excessive oxidation, triggered an inflammatory response, and activated caspase-3, resulting in apoptosis	[139]

Abbreviations: HK-2 human kidney-2; MAPK mitogen-activated protein kinase; UCP2 uncoupling protein 2.

## Abbreviations

AP allopurinol; APAP acetaminophen; APS astragalus polysaccharide; α-SMA α-smooth muscle actin; BMP-7 bone morphogenic protein 7; BSA bovine serum albumin; CAN chronic allograft nephropathy; CCl4 carbon tetrachloride; db/db diabetic obese; Cox2 cytochrome oxidase subunit 2; DFD Dahuang Fuzi Decoction; DNMTs DNA methyltransferases; Dox doxorubicin; EMT epithelial-mesenchymal transition; FN fibronectin; GFAT glutamine fructose 6-phosphate aminotransferase; GSK3b glycogen synthase kinase 3 beta; HEK human embryonic kidney; HGF hepatocyte growth factor; HK-2 human kidney-2; IgAN IgA nephropathy; IL-1 interleukin 1; ILK integrin-linked kinase; JNK c-JunNH2-terminal kinase; lncRNAs long noncoding RNAs; LPS lipopolysaccharide; MAPK mitogen-activated protein kinase; MMP-9 matrix metalloproteinase-9; MCGT1 mesangial cells transinfected with the human GLUT1 gene; MCLacZ mesangial cells transinfected with bacterial β-galactosidase; NF-κB nuclear factor kappa-B; Nrf2 the nuclear factor E2-related factor 2; NRK-49F normal rat kidney-49F; Nx nephrectomied; p38-MAPK p38 mitogen-activated protein kinase; PPARα peroxisome proliferator-activated receptor-α; RAC Rhubarb and astragalus capsule; RHL rhein lysinate; SAM senescence-accelerated mouse; SAMR1 senescence-resistant inbred strain 1; SAMP10 senescence-prone inbred strain 10; SD Sprague–Dawley; SIRT1 Sirtuin-1; STZ streptozotocin; TCMK-1 transformed C3H mouse kidney-1; TGF-β1 transforming growth factor- β1; TIMP1 TIMP metallopeptidase inhibitor 1; TLR4 toll-like receptor 4; TNF tumor necrosis factor; UUO unilateral ureteral obstruction; VCM vancomycin.
